# Unveiling the sedentary epidemic through insights from college students in Guangdong

**DOI:** 10.1038/s41598-025-19018-2

**Published:** 2025-10-08

**Authors:** Xiaoyu Tao, Xuelan Wu, Siying Zhuo, Jia fu, Ying Xiao, Yang Zhao, Junfeng Liao, Tian Zhong

**Affiliations:** 1 School of Management, Guangzhou College of Commerce, Guangzhou, 510500 China; 2 Hong Kong Public Health Technology Research Center, Hong Kong, 999077 China; 3 Research Center for Great Health and Socio-economic Development of Aging, Guangzhou College of Commerce, Guangzhou, 510500 China; 4 Faculty of Medicine, Macau University of Science and Technology, Taipa, Macao, China; 5 Faculty of Health Sciences, Zhuhai College of Science and Technology, Zhuhai, Guangdong China; 6 Faculty of Innovative Hospitality Management and Faculty of Creative Tourism and Intelligent Technologies, Macao university of Tourism, Mong-Há, Macao, China

**Keywords:** *Sedentary behavior*, *College students*, *Cross-sectional study*, Public health, Epidemiology

## Abstract

This study investigates the prevalence of sedentary behavior among college students in Guangdong Province, China, and identifies demographic factors associated with sedentary habits. A cross-sectional survey was conducted with 253 college students using a structured questionnaire. Participants’ sedentary behavior was assessed using the Sedentary Behavior Questionnaire (SBQ), with sedentary behavior defined as sitting for ≥7 hours per day. Data were analyzed using descriptive statistics, chi-square tests, t-tests, and analysis of variance (ANOVA). The majority of participants reported daily sitting times exceeding 10 hours, far surpassing the recommended threshold of 7 hours. Female students, those in lower academic years, and urban residents exhibited higher levels of sedentary behavior. Significant differences were observed in sedentary behavior based on academic year and place of residence, with senior students and those from rural areas reporting lower sitting times. Sedentary behavior is common among college students in Guangdong, with gender, academic year, and residence influencing sitting time.

**Supplementary Information:**

The online version contains supplementary material available at 10.1038/s41598-025-19018-2.

## Introduction

In the contemporary sociocultural milieu, sedentary behavior has become an omnipresent phenomenon, engendering substantial public health concerns due to its potential deleterious consequences on health. Extensive empirical investigations corroborate the pervasive nature of sedentary behavior across diverse demographic cohorts and geographical regions, with a discernible trajectory of escalating prevalence. A multitude of studies furnish robust evidence regarding the increasing ubiquity of this phenomenon. For example, an analysis of temporal variations in sedentary behavior among European Union adults from 2002 to 2017 revealed a pronounced surge in sedentary behavior rates across both genders. Specifically, the overall sedentary behavior rate within the EU population escalated from 49.3% in 2002 to 54.5% in 2017, with a marked increase in men from 51.2% to 55.8%, and women from 47.6% to 53.4%^1^. Besides, another systematic review indicates that the average daily sedentary time exceeds 8 hours across different regions^2^. In China, relevant studies have shown that over 28.8% of respondents exhibit sedentary behavior, with the prevalence increasing annually^3^.Similarly, during the COVID-19 pandemic, a study investigating adults with chronic diseases found that 71.5% of participants failed to meet recommended physical activity thresholds, with 62.7% exhibiting elevated risks of sedentary behavior^4^. These findings accentuate the ubiquitous and escalating nature of sedentary behavior in contemporary society.

The deleterious health consequences associated with sedentary behavior are manifold and cannot be trivialized. The modern sedentary lifestyle, compounded by suboptimal dietary practices, has precipitated a marked escalation in the incidence of atherosclerotic cardiovascular diseases (ASCVD), particularly in low- and middle-income nations^5,6^. Sedentary behavior is intrinsically linked to obesity, and public health initiatives targeting sedentary behavior are likely to influence health behaviors related to weight gain, systemic inflammation, and metabolic dysregulation, thereby exacerbating the prevalence of obesity, immunological impairments, and chronic pathologies^7^. Furthermore, the paucity of physical activity is a well-established risk factor for mental health disorders, including anxiety and depression. For instance, a UK-based investigation revealed that individuals experiencing a reduction in physical activity during the pandemic exhibited a concomitant exacerbation of depressive and anxiety symptoms^8^. Given the omnipresence of sedentary behavior and its potentially profound health repercussions, it is imperative to conduct focused research on sedentary behavior in specific demographic cohorts. The manifestation and ramifications of sedentary behavior may differ substantially across populations due to variations in physiological, psychological, and socio-environmental factors. For instance, rural pregnant women are disproportionately affected by obesity and sedentary behavior, potentially as a result of adverse perceptions of physical activity during pregnancy^9^. Similarly, children with congenital heart disease, irrespective of the severity of their condition or treatment modalities, are predisposed to sedentary lifestyles, with their patterns of physical activity and sedentary behavior typically becoming entrenched by the age of two and persisting through childhood^10^.

Among diverse populations, this study specifically targets the university student demographic, a cohort that exhibits distinctive patterns of sedentary behavior. It is well-documented that the majority of university students engage in sedentary behavior, often co-occurring with other health risk behaviors. A study encompassing 6,372 Chinese university students revealed that a significant proportion of students faced challenges related to sedentary behavior, insufficient physical activity, and poor dietary habits, with over 60% engaging in two or more health risk behaviors^11^. Moreover, the prolonged use of electronic devices represents a predominant manifestation of sedentary behavior among university students. A survey conducted among students from multiple Chinese universities revealed that 75.9% of students utilized electronic devices for a minimum of four hours per day^12^.

Significant strides have been made in understanding sedentary behavior and its associated factors among university students. From a health impact perspective, sedentary behavior is unequivocally linked to a range of health issues. Systematic reviews and meta-analyses have substantiated that sedentary behavior constitutes a significant risk factor for neck pain, with the risk intensifying in proportion to the duration of sedentary activity. However, the incidence of neck pain in student populations remains relatively lower compared to the workforce^13^. Moreover, sedentary behavior is associated with a plethora of adverse health outcomes, including diminished sleep quality, compromised cardiopulmonary function, elevated obesity indicators, and exacerbated symptoms of depression and anxiety^14–18^ Research has also illuminated the intricate interplay between sedentary behavior and factors such as physical activity, sleep, and emotional well-being. For instance, substituting sedentary behavior with moderate-intensity physical activity has been shown to alleviate depression and stress symptoms among university students, with gender-based differences in response^19^. Besides, recent research indicated that sedentary behavior was associated with cognitive function, memory, and attention, which can lead to improved academic performance^20^.

As for the specific case of university students in Guangdong, recent data on sedentary behavior and its influencing factors remain unavailable. However, it is reasonable to infer that university students in Guangdong may face similar challenges related to sedentary behavior. Given the cultural, lifestyle, and academic pressures unique to this region, it is crucial to conduct focused research on university students in Guangdong to better understand their sedentary behavior patterns and contributing factors. Such research will enable the development of tailored interventions aimed at mitigating sedentary behavior in this population.

The findings of this study will provide valuable insights into sedentary behavior among university students in Guangdong Province and offer essential data to inform the creation of targeted interventions designed to reduce sedentary behavior within this group.

## Methods

### Research design

A cross-sectional research design was employed in this study to investigate the current status of sedentary behavior and its associated factors among college students in Guangdong Province. Data were collected through questionnaires to comprehensively understand the patterns of sedentary behavior and their association with demographic characteristics.

### Sample size calculation

The sample size was calculated using the cross-sectional study formula^10^: *n*=$\frac{{Z}^{2}\cdot p\cdot (1-p)}{{e}^{2}}$, where Z is the Z-value from the standard normal distribution corresponding to the desired confidence level (1.96 for a 95% confidence level), *p* is the expected proportion (approximately 70% based on prior studies), and e is the margin of error (set at 8%). Substituting the values into the formula yielded n≈248.

### Study subjects

The study subjects were 253 college students from a university in Guangdong Province, including 125 females and 128 males. The participants covered various majors (Literature, Arts, Business and Economics, Medicine, Science and Engineering) and different academic years (freshman to fifth-year students). Individuals who were unable to complete the questionnaire due to health reasons were excluded from the study.

### Sampling method

A multi-stage sampling method was employed in this study. In the first stage, several colleges were randomly selected from all colleges in Guangdong Province, based on college size and major distribution. In the second stage, several classes were randomly selected within the chosen colleges. In the third stage, a certain number of students were randomly selected within the chosen classes, based on student ID numbers, to ensure the representativeness and diversity of the sample.

### Data collection

Data collection was conducted in March 2025 using a questionnaire survey. The questionnaire included components such as demographic information (gender, major, academic year, vacation residence, smoking status, and drinking status), body mass index (BMI) calculated using self-reported height and weight and categorized according to the World Health Organization standards^21^. Sedentary behavior was assessed using the Chinese version of the Sedentary Behavior Questionnaire (SBQ), which has been validated for use among Chinese university students and demonstrated high internal consistency (Cronbach’s alpha = 0.967)^22^. The SBQ was used to record sitting time on weekdays and weekends, with prolonged sedentary behavior defined as sitting for ≥7 hours per day^23,24^. All data related to sedentary behavior and other variables were self-reported by the participants.

### Variable definition

The dependent variable was sedentary behavior (sitting time ≥7 hours/day), while the independent variables included demographic characteristics (gender, major, academic year, vacation residence, smoking status, drinking status) and BMI categories.

### Data analysis

Data analysis was performed using SPSS 26.0. Descriptive statistics were calculated for each variable, including frequencies, percentages, means, and standard deviations. Chi-square tests were used to compare differences in BMI categories across demographic characteristics. t-test and analysis of variance (ANOVA) were employed to compare differences in sitting time across groups, with the LSD (Least Significant Difference) method used for post-hoc tests if the ANOVA showed significant differences. All statistical tests were set at a significance level of *P* < 0.05.

### Research ethics

This study was approved by the institutional ethics committee of Hong Kong Public Health Technology Research Center (HKPHTRC), with the ethics approval number PHTR/25/006-0010. Informed consent was obtained from all participants before data collection, ensuring they fully understood the study purpose and process and participated voluntarily. All procedures were conducted in accordance with the ethical standards of the Hong Kong Public Health Technology Research Center (HKPHTRC) and with the 1964 Helsinki Declaration and its later amendments

## Results

### Demographic characteristics

The study included 253 participants, with 125 females and 128 males. Nearly 20% of participants majored in Medical and the mean value of BMI of the students population was within normal range (22.12 ± 3.20 kg/m^2^). The details as shown in Table 1.

**Table 1:** Characteristics of participants stratified by gender.

**Variable(%)**	**Total**	**Male**	**Female**
**Specialty**			
Literature	73(28.9)	28(21.9)	45(36.0)
Arts	31(12.3)	20(15.6)	11(8.8)
Business and Economics	43(17.0)	18(14.1)	25(20.0)
Medicine	53(20.9)	31(24.2)	22(17.6)
Science and Engineering	53(20.9)	31(24.2)	22(17.6)
**Grade**			
Freshman	65(25.7)	32(25.0)	33(26.4)
Sophomore	68(26.9)	38(29.7)	30(24.0)
Junior	60(23.7)	21(16.4)	39(31.2)
Senior	48(19.0)	28(21.9)	20(16.0)
Fifth-year undergraduate	12(4.7)	9(7.0)	3(2.4)
**Vacation residence**			
Rural areas	94(37.2)	43(33.6)	51(40.8)
urban areas	159(62.8)	85(66.4)	74(59.2)
**Smoking status**			
Smoker	70(27.7)	61(47.7)	9(7.2)
Non-smoker	183(72.3)	67(52.3)	116(92.8)
**Drinking status**			
Drinker	102(40.3)	64(50.0)	38(30.4)
Non-drinker	151(59.7)	64(50.0)	87(69.6)
**BMI Categories**			
underweight	32(12.6)	7(5.5)	25(20.0)
normal weight	154(60.9)	76(59.4)	78(62.4)
overweight	57(22.5)	40(31.3)	17(13.6)
obese	10(4.0)	5(3.9)	5(4.0)

The majority participants had a normal BMI, but 26.5% were classified as overweight or obese. There was a significant difference in BMI categories between genders (*P*<0.001) and smoking status (*P*=0.010). Male students had a higher prevalence of being underweight. No significant difference in BMI categories were found based on academic specialty. The details as shown in Table 2.

**Table 2:** Demographics by BMI categories and the analysis results.

**Characteristics**	**BMI categories**				**ν**	**χ **^**2**^**-value**
	**Underweight**	**Normal weight**	**Overweight**	**Obese**		
**Gender**					3	19.40^***^
Male	7	76	40	5		
Female	25	78	17	5		
**Specialty**					12	13.48
Literature	11	45	15	2		
Arts	4	12	12	3		
Business and Economics	5	31	6	1		
Medicine	7	33	12	1		
Science and Engineering	5	33	12	3		
**Grade**					12	14.59
Freshman	10	38	15	2		
Sophomore	4	41	17	6		
Junior	8	38	12	2		
Senior	10	28	10	0		
Fifth-year undergraduate	0	9	3	0		
**Vacation residence**					3	1.85
Rural areas	14	53	22	5		
urban areas	18	101	35	5		
**Smoking status**					3	11.44^*^
Smoker	4	38	23	5		
Non-smoker	28	116	34	5		
**Drinking status**					3	0.42
Drinker	13	60	25	4		
Non-drinker	19	94	32	6		

^*^P<0.05, ***P<0.001.

### Participants’ sedentary behavior

#### Overview of sitting time by participant characteristics

Notably, except for fifth-year students, all subgroups reported prolonged sitting time, with total, weekday, and weekend sitting durations exceeding 10 hours. Females reported slightly higher average sitting times on both weekdays (11.24 ± 4.49 min/day) and weekends (11.95 ± 5.37 min/day) compared to males (10.90 ± 4.78 and 11.77 ± 5.50, respectively). Correspondingly, a higher proportion of females (83.20%) engaged in prolonged sedentary behavior than males (78.13%). Besides, Students majoring in Science and Engineering and Arts reported the highest average sitting times (11.27 ± 4.06 and 11.14 ± 4.27 min/day, respectively) and the highest prevalence of prolonged sedentary behavior (84.91% and 83.87%). In contrast, Medicine students had the lowest average sitting time (11.06 ± 4.58 min/day) and the lowest prevalence (71.70%). Additionally, the proportion of students with sitting time ≥7 hours/day was high across all groups, consistently surpassing 70%. Detailed are presented in Table 3.

**Table 3:** Sitting time and prevalence of prolonged sedentary behavior (≥7 h/day) stratified by participant characteristics.

**Characteristics**	**Total sitting time of SB**^**a**^**(min/day)**		**Average total sitting time of SB**^**a**^**(min/day)**	**Sitting time≥ 7 h/day (%)**
	**Weekdays**	**Weekends**		
**Gender**				
Male	10.90±4.78	11.77±5.50	11.15±4.42	78.13%
Female	11.24±4.49	11.95±5.37	11.44±4.21	83.20%
**Specialty**				
Literature	11.48±4.80	11.60±5.00	11.51±4.34	83.56%
Arts	11.03±4.21	11.40±6.44	11.14±4.27	83.87%
Business and Economics	11.30±4.65	11.47±5.69	11.35±4.44	79.07%
Medicine	10.38±5.03	12.74±5.19	11.06±4.58	71.70%
Science and Engineering	11.02±4.27	11.91±5.47	11.27±4.06	84.91%
**Grade**				
Freshman	11.25±4.58	12.05±5.93	11.48±4.41	80.00%
Sophomore	11.71±4.21	13.57±4.97	12.24±3.81	86.76%
Junior	10.91±4.34	10.63±5.24	10.83±4.00	85.00%
Senior	11.31±4.58	11.39±5.35	11.27±4.82	79.17%
Fifth-year undergraduate	6.40±4.73	9.15±6.02	7.18±4.80	33.34%
**Vacation residence**				
Rural areas	9.76±4.75	11.04±5.24	10.12±4.37	71.27%
urban areas	11.84±4.40	12.34±5.49	11.99±0.35	86.16%
**Smoking status**				
Smoker	10.65±4.50	10.75±4.54	10.98±6.38	75.71%
Non-smoker	11.23±4.68	12.20±4.99	11.50±4.21	82.51%
**Drinking status**				
Drinker	10.79±4.70	11.42±5.55	10.97±4.33	80.39%
Non-drinker	11.25±4.59	12.16±5.34	11.52±4.30	80.79%

^a^ SB:Sedentary Behaviour.

#### Total sitting time

Significant differences were observed in grade (*P*=0.004) and vacation residence (*P* < 0.001). Specifically, Fifth-year undergraduate reported significantly lower total sitting time compared to all other grades, as confirmed by post-hoc analysis (*P*<0.05). No significant differences were found across other characteristics. These findings are further detailed in Table 4.

**Table 4:** Total sitting time stratified by participant characteristics.

**Characteristics**	**Total sitting time**	**t/F**	**Post-hoc test(LSD)**
**Gender**		0.54	/
Male	11.15±4.42		
Female	11.44±4.21		
**Specialty**		0.10	/
Literature	11.51±4.34		
Arts	11.14±4.27		
Business and Economics	11.35±4.44		
Medicine	11.06±4.58		
Science and Engineering	11.27±4.06		
**Grade**		3.93^**^	Grade 5<Grade 1-4 (P<0.01)
Freshman	11.48±4.41		
Sophomore	12.24±3.81		
Junior	10.83±4.00		
Senior	11.27±4.82		
Fifth-year undergraduate	7.18±4.80		
**Vacation residence**		−3.39^**^	/
Rural areas	10.12±4.37		
urban areas	11.99±0.35		
**Smoking status**		−1.25	/
Smoker	10.98±6.38		
Non-smoker	11.50±4.21		
**Drinking status**		−0.98	/
Drinker	10.97±4.33		
Non-drinker	11.52±4.30		

^**^P<0.01.

#### Weekday sitting time

Comparative analyses of weekday sitting time revealed significant differences across grade (*P* = 0.007) and vacation residence (*P* < 0.001), based on t-tests and one-way ANOVA with LSD post-hoc tests. Similar to total sitting time, Fifth-year undergraduate students reported significantly lower weekday sitting time compared to students in other academic years**.** No statistically significant differences were found across other participant characteristics. The results are summarized in Table 5.

**Table 5:** Weekday sitting time stratified by participant characteristics.

**Characteristics**	**Weekday Sitting Time**	**t/F**	**Post-hoc test**
**Gender**		0.58	/
Male	10.90±4.78		
Female	11.24±4.49		
**Specialty**		0.46	/
Literature	11.48±4.80		
Arts	11.03±4.21		
Business and Economics	11.30±4.65		
Medicine	10.38±5.03		
Science and Engineering	11.02±4.27		
**Grade**		3.593^**^	Grade 5<Grade 1-4 (P<0.01)
Freshman	11.25±4.58		
Sophomore	11.71±4.21		
Junior	10.91±4.34		
Senior	11.31±4.58		
Fifth-year undergraduate	6.40±4.73		
**Vacation residence**		−3.55^***^	/
Rural areas	9.76±4.75		
urban areas	11.84±4.40		
**Smoking status**		−0.88	/
Smoker	10.65±4.50		
Non-smoker	11.23±4.68		
**Drinking status**		−0.78	/
Drinker	10.79±4.70		
Non-drinker	11.25±4.59		

^**^P<0.01, ***P<0.001.

#### Weekend sitting time

Significant differences in weekend sitting time were observed across grade (*P* =0.009), as indicated by ANOVA with LSD post-hoc tests. Specifically, Sophomore reported significantly higher weekend sitting time compared to Junior, Senior, and Fifth-year undergraduate students. No significant differences were found for other participant characteristics. Results are shown in Table 6.

**Table 6:** Weekend sitting time stratified by participant characteristics.

**Characteristics**	**Weekend Sitting Time**	**t/F**	**Post-hoc test**
**Gender**		0.28	/
Male	11.77±5.50		
Female	11.95±5.37		
**Specialty**		0.51	/
Literature	11.60±5.00		
Arts	11.40±6.44		
Business and Economics	11.47±5.69		
Medicine	12.74±5.19		
Science and Engineering	11.91±5.47		
**Grade**		3.46^**^	Grade 2>Grade 3-5 (*P*<0.05)
Freshman	12.05±5.93		
Sophomore	13.57±4.97		
Junior	10.63±5.24		
Senior	11.39±5.35		
Fifth-year undergraduate	9.15±6.02		
**Vacation residence**		−1.86	/
Rural areas	11.04±5.24		
urban areas	12.34±5.49		
**Smoking status**		−1.61	/
Smoker	10.75±4.54		
Non-smoker	12.20±4.99		
**Drinking status**		−1.07	/
Drinker	11.42±5.55		
Non-drinker	12.16±5.34		

^**^P<0.01.

## Discussion

Research findings indicate that university students exhibit pervasive and prolonged sedentary behavior, with both weekday and weekend durations surpassing the recommended threshold of 7 hours per day. Empirical evidence demonstrates a significant correlation between sedentary behavior in university students and health-related risk factors, particularly during weekends^25^. Furthermore, academic schedules have been shown to significantly influence sedentary behavior and physical activity patterns. Specifically, each additional hour of class time corresponds to a 9-minute increase in sedentary behavior, while moderate-to-vigorous physical activity only increases by 54 seconds^26^. These findings underscore the imperative of reducing sedentary behavior among university students and advocate for intervention strategies that account for academic schedules and weekend activities.

Research also highlights gender disparities in sedentary behavior, with female students exhibiting longer sedentary durations than their male counterparts, potentially attributable to divergent lifestyle patterns and study habits. Gender-specific responses to mindfulness training reveal that females exhibit greater reductions in negative emotions and enhancements in mindfulness and self-compassion following such interventions^27^. This indicates that mindfulness practices may serve as an effective strategy for mitigating the psychological repercussions of sedentary behavior in female students.

Analysis of sedentary behavior across academic years reveals that senior students (e.g., fifth-year students) exhibit significantly lower sedentary durations compared to their junior counterparts. This trend may be attributable to increased participation in extracurricular activities and internships among senior students^28^. Geospatial variations further influence sedentary behavior, with urban students demonstrating longer sedentary durations than rural students. This disparity may stem from reduced recreational spaces in urban environments and a faster-paced lifestyle that predisposes students to static leisure activities^29^. Moreover, urban students exhibit higher physical activity levels on weekends but reduced activity during weekdays, likely due to academic pressures and transportation constraints^29^.

The relationship between sedentary behavior and health-risk behaviors such as smoking and hazardous drinking has garnered significant attention. A systematic review of cohort studies examined the association between various health behaviors, including sedentary behavior, alcohol intake, and smoking, and academic performance among tertiary education students. Although the number of studies specifically on sedentary behavior was limited (only 2 out of 34 included studies), this review highlights the importance of considering multiple health - risk behaviors in the university student population. It found consistent negative associations between alcohol intake and academic performance, suggesting that health - risk behaviors can have far - reaching consequences beyond just physical health^30^. These findings accentuate the necessity of promoting physical activity among youth to mitigate sedentary behavior and its associated health risks.

The findings of this study contribute to the global discourse on sedentary behavior by highlighting the prevalence and patterns among university students in an under-researched region. Our results emphasize the importance of considering academic schedules, gender differences, and urban-rural disparities when designing interventions to reduce sedentary behavior. These insights may inform policy initiatives aimed at promoting healthier lifestyles among young adults globally, particularly in educational settings. By identifying specific factors associated with sedentary behavior, our study provides a foundation for targeted interventions that can be adapted to various contexts, thereby supporting broader public health efforts to address this growing concern.

Notwithstanding these insights, the study possesses certain limitations. The cross-sectional design precludes establishing causality between sedentary behavior and health risks. Additionally, sedentary behavior is influenced by a range of factors, including psychological (e.g., depression, life satisfaction), cognitive and motivational (e.g., attitudes, social support, self-efficacy), and sociodemographic factors (e.g., socioeconomic status, occupational type)^23–25^. The sample was also limited to one university in Guangdong Province, which may restrict generalizability. Future research should broaden sampling to include diverse geographical and institutional contexts to enhance external validity.

## Conclusion

This cross-sectional study reveals the prevalence and correlates of sedentary behavior among college students in Guangdong Province, showing that most students engage in over 10 hours of daily sedentary time, significantly exceeding the recommended 7-hour threshold and being closely associated with factors such as gender, academic year, and place of residence. Notably, female students, those in lower academic years, and urban residents exhibited higher levels of sedentary behavior, reflecting challenges in promoting healthy lifestyles. The study highlights the detrimental effects of sedentary behavior on physical health (e.g., obesity and cardiovascular risk) and mental well-being, advocating for health education, improved sports facilities, and active lifestyle promotion to reduce sedentary time. However, the cross-sectional design limits causal inferences, and the relatively small sample size may restrict generalizability. Future research should expand sampling frameworks and investigate the nuanced impacts of different types of sedentary behavior to further inform health management strategies for college students.

## Supplementary Information

Supplementary Information.

## Data Availability

The raw data supporting this study are provided in Supplementary File.
